# Study on nonlinear dynamics characteristics of dual speed dual clutch transmission system based on bond graph

**DOI:** 10.1016/j.heliyon.2023.e20862

**Published:** 2023-10-10

**Authors:** Jiangming Wu, Hongzhi Yan, Shuangqi Liu, De Ni

**Affiliations:** a School of Mechanical and Electrical Engineering, Central South University, Changsha, 410083, China; b The State Key Laboratory of High-Performance and Complex Manufacturing, Central South University, Changsha, 410083, China; c AECC Hunan Aviation Powerplant Research Institute, Zhuzhou, 412002, China

**Keywords:** Bond graph, Compound planetary gear train, Clutch, Shift dynamic model

## Abstract

The variable speed transmission system employs compound planetary gear trains and clutches. Compared to traditional aviation transmission systems, the added control parts in the variable transmission system result in a stronger coupling between components and more complex dynamic response characteristics. Modeling using the central mass method is complex and abstract. This study proposes a bond graph-based method for modeling and analyzing the dynamic behavior of the variable transmission system. The transmission system is divided into multiple component-level real-time dynamic models, and obtain a nonlinear multi-energy domain shift dynamic models based on energy flow paths coupling. The mathematical model is numerically solved using the Runge-Kutta method. Simulation results show that there is a delay in the speed response during the shifting process. Further analysis reveals that this “give and take” phenomenon is related to the mechanical structure and passive working principle of the one-way clutch. Subsequently, the time-domain response of the output speed under the hydraulic loading characteristic curves of linear, exponential, S-shaped, and composite functions, as well as the clutch torque, were studied. The impact of different hydraulic loading durations based on the exponential curve on component speed and torque was also analyzed. The simulation results provide a theoretical basis for designing low-shift impact, high-reliability, and high-performance variable transmission systems.

## 1 Introduction

Dynamic modeling is an important task in research on the control and optimization of transmission. The dynamic study of the transmission system is made more challenging by the usage of compound planetary gear trains and clutches. However, from the control perspective, the dynamical model for transmission should be simple and intuitive, while also reflecting the main dynamic responses and inherent characteristics of transmission. Therefore, an intuitive and accurate dynamic model helps understand the power transmission in the system, reduces the workload of the hardware system, and improves the efficiency of the simulation and optimization design.

The variable speed transmission system studied in this study is applicable to aviation transmission, which can achieve different propeller tip speed in high speed cruise state and hovering state of helicopters [1]. Compared with the constant rotor speed scheme, by changing the main rotor speed to react to a variety of flight circumstances, it is effective in solving rotor noise, breaking forward flight speed, increasing payload and range, reducing fuel consumption and maintenance costs, etc [2]. Variable speed rotor technology has become one of the key technologies for future military heavy helicopters and advanced composite helicopters [3].

Newton Method [4,5], Lagrange equation [6,7], and bond graph methods [8,9] are often used to build models of transmission systems to transform a physical system into a differential equation. Compared with other methods, the bond graph theory can help researchers gain insights into the changes of multiple state variables in the system under different states reflecting the performance and dynamic characteristics of the system. Previous studies [10,11] have introduced the modeling of dynamic systems and components in different energy fields, and creatively applied bond graphs to analyze transmission system dynamics. In a study by Ranogajec [12], the shift characteristics of a AT gearbox were analyzed using the bond graph method. Ivanovic [13] summarized the bond graph model of common planetary gear set. The bond graph method has also been used to simulate an entire vehicle model [14,15]. Hu [16] analyzed the transmission efficiency under all possible power flows based on a bond graph model, in order to design and optimize low-impact control strategies. Furthermore, based on the standard definition of causality for a bond graph, Li [17] designed a standardized modeling process of gearbox, but did not pay attention to clutch. Liu [18] constructed a double planetary gear transmission dynamics model including the dynamics between planetary bearing roller-cage, planetary gear-bearing-carrier, and roller-cage, analyzed the inherent characteristics of the wheel system and the effect of speed variation on vibration, and verified the validity of the model through experiments. In the literature [19], for the problem that it is difficult to analyze the vibration difference of rotor in different axial positions by rigid dynamic system model, the flexible shaft segment is coupled with gear meshing unit and bearing unit and solved by finite element method.

The majority of the current study focuses on the investigation of the gear system's dynamic response law in steady state without thoroughly examining the impact of external excitation; and because of the complex structure of the gear transmission system and the strong coupling relationship between multiple pairs of gears and multiple clutches, the commonly used modeling methods have strict requirements on the structure of the transmission system or lack of control system-oriented design considerations; when there are non-wheel system components in the system, clear coupling methods and principles are not provided, and the modeling process is not universal and adaptable. To address this issue, this study aims to introduce a control-oriented and general bond-graph-based method for the dynamic modeling of transmission systems comprising a compound planetary gear train and clutches. Based on a bond graph model of two-speed double clutch (TSDC) transmission, the impact of the clutch working state on the dynamic characteristics of the shifting process is investigated.

## 2 Working principle of TSDC transmission

**Fig. 1:**
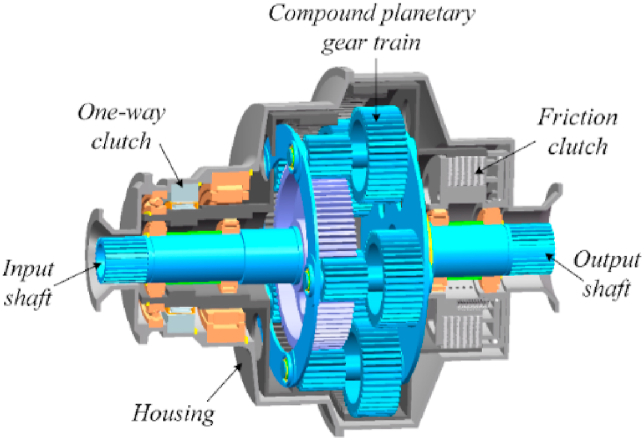
Three-dimensional model of TSDC transmission.

**Fig. 2:**
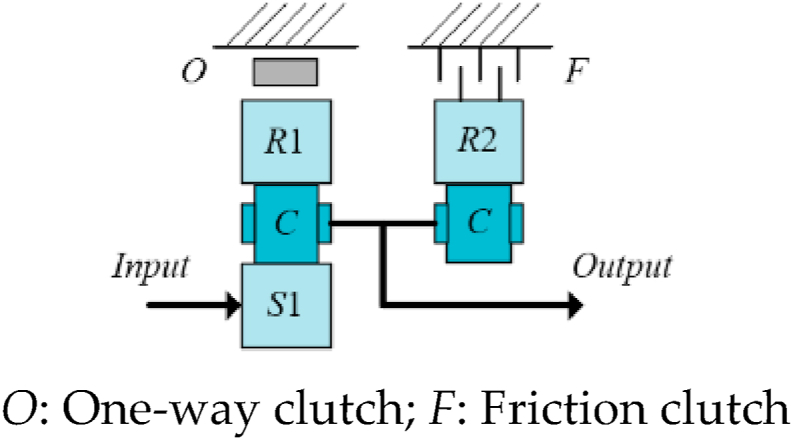
Schematic of TSDC transmission.

Fig. 1 shows a three-dimensional model of the TSDC transmission structure. Fig. 2 shows the stick diagram for TSDC transmission.

(1) High-speed gear: friction clutch is engaged, and ring gear R2 is firmly connected to the flange, which is indirectly fixed to the body of the gearbox and cannot be rotated. Ring gear R1 and sun gear S rotate in a clockwise direction.
(2) Low-speed gear: the friction clutch is disengaged, ring gear R2 can rotate freely, the one-way clutch completely locks ring gear R1 to the gearbox body, and the output shaft rotates at a low speed.

## 3 Compound dynamic model for TSDC transmission

### 3.1 Compound planetary gear train

**Fig. 3:**
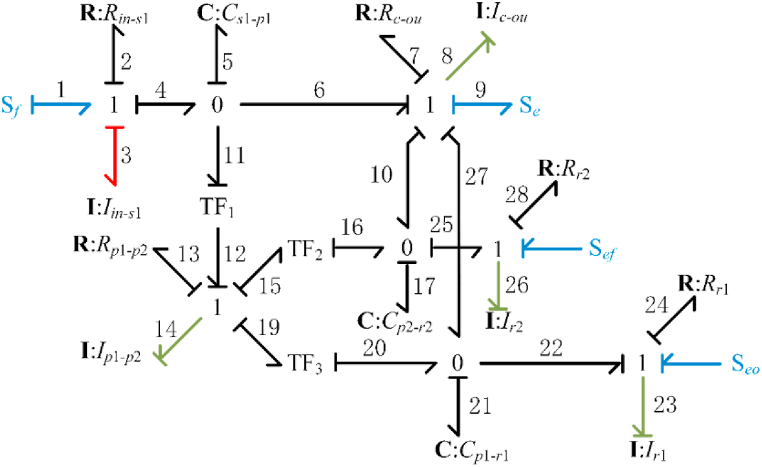
Enhanced bond graph model of the full-order gear train.

Fig. 3 illustrates the enhanced bond graph model of the TSDC transmission, which only includes the dominant gear set elements. Other elements, such as gear backlash and friction, are left out but can be included to the model as lumped input/output factors [20]. The meaning of each component of the bond graph is listed in Table 1, where the junction points 0 and 1 represent the independent parts and interactions between parts, respectively [21].

**Table 1:** Meaning of each element in the bond graph.

Symbol	Meaning
*S*_*f*_, *S*_*e*_, *S*_*ef*_, and *S*_*eo*_	Flow sources acting on the input shaft, effort source acting on the output shaft, and effort sources from the friction clutches and one-way clutch, respectively.
*R*_*in-s*_, *R*_*c-ou*_, *R*_*p1-p2*_, *R*_*r1*_, and *R*_*r2*_	Damping on the input shaft (sun gear S), output shaft (carrier C), double planetary gears P1–P2, ring gear R1, and ring gear R2, respectively.
*I*_*in-s*_, *I*_*c-ou*_, *I*_*p1-p2*_, *I*_*r1*_, and *I*_*r2*_	Moment of inertia of input shaft (sun gear S), output shaft (planetary carrier C), double planetary gears P1–P2, ring gear R1, and ring gear R2, respectively.
*C*_*s-p1*_, *C*_*p1-r1*_, *and C*_*p2-r2*_	Compatibility elements related to the average meshing stiffness between the sun gear S and the planet gear P1, the average meshing stiffness between the planet gear P1 and the ring gear R1, and the average meshing stiffness between the planet gear P2 and the ring gear R2 and the stiffness of the corresponding shaft.
TF_1_, TF_2_, and TF_3_	Converter with size zp1/zs, -zr2/zp2, and -zr1/zp1, respectively.

The capacitive element *C* and the inertial element *I* with integral causality were considered as state variables in Fig. 3. The equation of state is obtained from the bond composition law equation.

State variable: $X={\left[\begin{array}{ccccccc} q_{5} & p_{8} & p_{14} & q_{17} & q_{21} & p_{23} & p_{26} \end{array}\right]}^{T}$.

Input variable: $U={\left[\begin{array}{cccc} S_{f} & S_{e} & S_{eo} & S_{ef} \end{array}\right]}^{T}$.

The effort variables *e*, flow variables *f*, generalized displacements *p*, and generalized momentum *q* in the bond diagram correspond to the torque *τ*, angular velocity *ω*, angular displacement *θ*, and angular momentum *L* in TSDC transmission.

(1) $$\left\{ \begin{array}{cc} {\dot{q}}_{5}=S_{f}-\frac{1}{I_{8}}p_{8}-{\text{TF}}_{1}\frac{1}{I_{14}}p_{14} & {\dot{q}}_{21}=\frac{1}{I_{8}}p_{8}+\frac{1}{{\text{TF}}_{3}}\frac{1}{I_{14}}p_{14}-\frac{1}{I_{23}}p_{23}\\ {\dot{p}}_{8}=\frac{1}{C_{5}}q_{5}-R_{7}\frac{1}{I_{8}}p_{8}-\frac{1}{C_{17}}q_{17}-\frac{1}{C_{21}}q_{21}-S_{e} & {\dot{p}}_{23}=\frac{1}{C_{21}}q_{21}-R_{24}\frac{1}{I_{23}}p_{23}+S_{eo}\\ {\dot{p}}_{14}={\text{TF}}_{1}\frac{1}{C_{5}}q_{5}-R_{13}\frac{1}{I_{14}}p_{14}-\frac{1}{{\text{TF}}_{2}}\frac{1}{C_{17}}q_{17}-\frac{1}{{\text{TF}}_{3}}\frac{1}{C_{21}}q_{21} & {\dot{p}}_{26}=\frac{1}{C_{17}}q_{17}-R_{28}\frac{1}{I_{26}}p_{26}+S_{ef}\\ {\dot{q}}_{17}=\frac{1}{I_{8}}p_{8}+\frac{1}{{\text{TF}}_{2}}\frac{1}{I_{14}}p_{14}-\frac{1}{I_{26}}p_{26} & \end{array}\right.$$

where $C_{5}=1/k_{s1p1},C_{17}=1/k_{p2r2},C_{21}=1/k_{p1r1},I_{8}=I_{cou},I_{14}=I_{p1\_p2},I_{23}=I_{r1},I_{26}=I_{r2}$. Equation (1) can be simplified to Equation (2).

(2) $$\dot{X}=AX+BU$$

Here, matrices A and B are respectively composed of the coefficients of the corresponding variables.

The derived gear set model has a nonlinear structure. This nonlinearity is mainly due to the nonlinear effort sources (S_*ef*_ and S_*eo*_), which explains the change it the torque of the friction clutch and the one-way clutch.

### 3.2 Clutch friction model

In transmission systems, the wet clutch is a vital component [22]. From a microscopic point of view, the micro-convex deformation resistance, furrow resistance, and adhesive friction resistance on the surface of the friction plate result in a friction force [23]. During engagement, the friction coefficient does not remain constant but instead has time-varying characteristics, it can be expressed using the Stribeck curve [24], such as Equation (3).

(3) $$\mu \left(t\right)=\mu_{c}+\left(\mu_{s}-\mu_{c}\right)\exp\left(-\delta \left|\omega_{rel}\right|\right)$$

where *μ*_*c*_ is the dynamic friction coefficient, *μ*_*s*_ is the static friction coefficient, *C*_1_ is the viscous friction coefficient, *δ* is the Stribeck coefficient, and *ω*_*r*el_ is the relative speed between ring gear R2 and the housing. The calculation of the friction torque transmitted during the engagement of the wet friction clutch is shown in Equation (4).

(4) $$T_{fslip}\left(\omega_{rel},p\right)=NAr_{m}p\left(t\right)\mu \left(t\right)\text{sgn}\left(\omega_{rel}\right)$$

The maximum friction torque that can be driven by the clutch is calculated using Equation (5).

(5) $$T_{fstick}=NAr_{m}\mu_{s}p\left(t\right)\text{sgn}\left(\omega_{rel}\right)$$

When *ω*_*rel*_ = 0 rad/s, the positive and negative values of the friction torque in the model are uncertain. Therefore, Karnopp friction torque model [25] was developed, assuming a range of approximately |ε| and external torque *T*_*e*_, the Karnopp friction torque model is shown in Equation (6).

(6) $$\tau_{f}=\left\{ \begin{array}{cc} T_{fslip}\left(\omega_{rel},p\right) & \omega_{rel}>\left|\varepsilon \right|\\ T_{fstick}\text{sgn}\left(T_{e}\right) & \omega_{rel}\leq \left|\varepsilon \right|\&\left|T_{e}\right|>T_{fstick}\\ T_{e} & \omega_{rel}\leq \left|\varepsilon \right|\&\left|T_{e}\right|\leq T_{fstick} \end{array}\right.$$

**Fig. 4:**
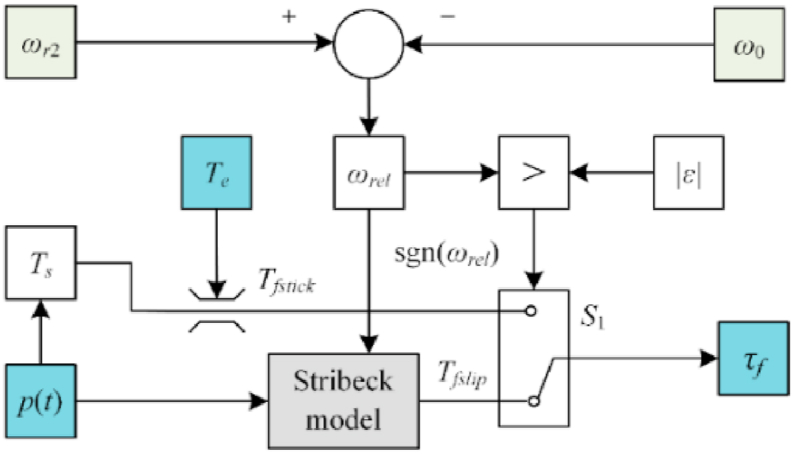
Block diagram of Karnopp friction model for hydraulic clutch.

Fig. 4 shows the Karnopp friction model of the hydraulic clutch based on the Karnopp friction model. Here, the torque of the friction clutch *τ*_*f*_ is the effort source, *S*_*ef*_, shown in Fig. 3.

### 3.3 One-way clutch nonlinear stiffness model

A one-way clutch is a type of passive mechanical clutch that is restricted by its structural properties to only spinning in one direction [26,27]. Modeling the one-way clutch as a nonlinear spring with discontinuous stiffness, zero stiffness in the disengaged state and finite linear stiffness in the engaged state. The angle difference the driving and driven ends of the clutch under working torque may be measured using the finite element method, thereby obtaining the fitted linear stiffness value of the forward engagement. Typically, the dynamic model might employ the relative velocity [28] or relative displacement angle [29] between the driving and driven ends of the clutch as a criterion for evaluating the mathematical model's operational condition. Here, a displacement angle difference-based nonlinear stiffness model is used, expressed as Equation (7).

(7) $$\tau_{o}=\left\{ \begin{array}{ccc} K_{o}\left(\theta_{r1}-\theta_{o}\right)+C_{o}\left({\dot{\theta }}_{r1}-{\dot{\theta }}_{o}\right) & & \theta_{r1}>\theta_{o}\\ 0 & & \theta_{r1}\leq \theta_{o} \end{array}\right.$$

### 3.4 Coupling of sub-dynamic models

**Fig. 5:**
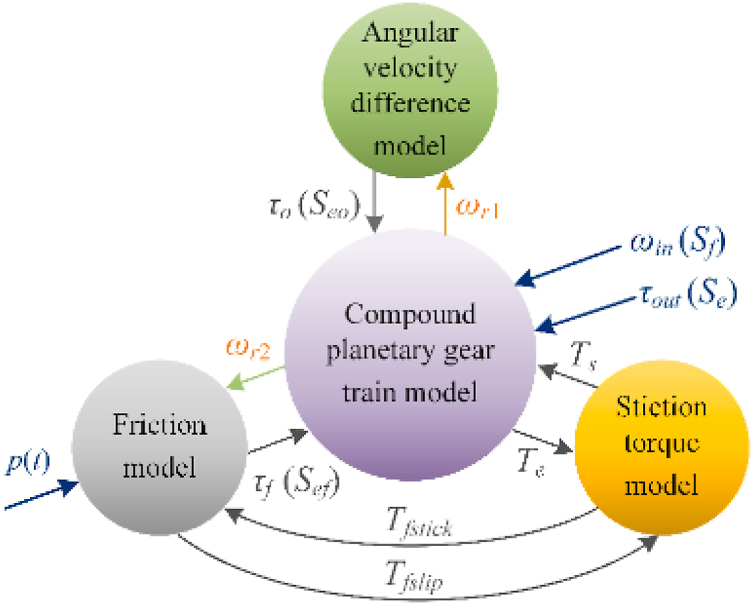
Component level dynamic coupling model of the TSDC transmission system based on energy flow relationship.

Considering the structural characteristics and operating principle of TSDC transmission, the abovementioned three sub-dynamic models are coupled. Fig. 5 shows the coupling relationship among the three models.

Under the external excitation of input speed *ω*_*in*_ and load torque *τ*_*out*_, *ω*_*r*1_, and *ω*_*r*2_, are transmitted to the wet friction clutch and one-way clutch, respectively. Under the excitation of hydraulic pressure *p*(*t*). The friction torque *τ*_*f*_ is transmitted to ring gear R2. The torque *τ*_*o*_ is transmitted to planetary gear train under the excitation of the rotating speed *ω*_*r*1_.

## 4 Simulation and analysis of TSDC transmission dynamic model

### 4.1 Parameter setting

Sun gear S possesses a power source connected that demonstrates the engine. As the rotating speed, an input rotating speed that reaches 628 rad/s within 2 s and then remains constant was adopted. An effort source that represents the load torque is attached to the carrier. The torque loads are determined based on the rotating speeds. At 30 s, the hydraulic system starts loading the hydraulic pressure, and it completes the linear loading of the rated hydraulic pressure within 0.5 s. When the relative speed between ring gear R2 and the housing is less than 0.001 rad/s, the friction clutch's torque is the same as the external torque. Table 2 lists other parameters utilized in the simulation.

**Table 2:** Numerical simulation parameter settings for TSDC transmission [30].

Subsystem	Parameter	Value	Unit
Compound planetary gear train	Meshing torsional stiffness *K*	288800	Nm/rad
	Rotational damping *C*	0.001	N·m·s/rad
Friction clutch	Nominal hydraulic pressure *P*_*a*_	0.8	MPa
	Number of friction plates *N*	8	/
	Inner radius of friction plate *r*_1_	77.5	mm
	Outer radius of friction plate *r*_2_	110	mm
	Piston cavity area *A*	0.028	m2
	Dynamic friction coefficient *μ*_*c*_	0.1	/
	Stribeck coefficient *δ*	0.02	
	Static friction coefficient *μ*_*s*_	0.12	/
One-way clutch	Torsional stiffness *K*_*o*_	28500	Nm/rad
	Torsional damping *C*_*o*_	0.001	N·m·s/rad

### 4.2 Simulation results and analysis

**Fig. 6:**
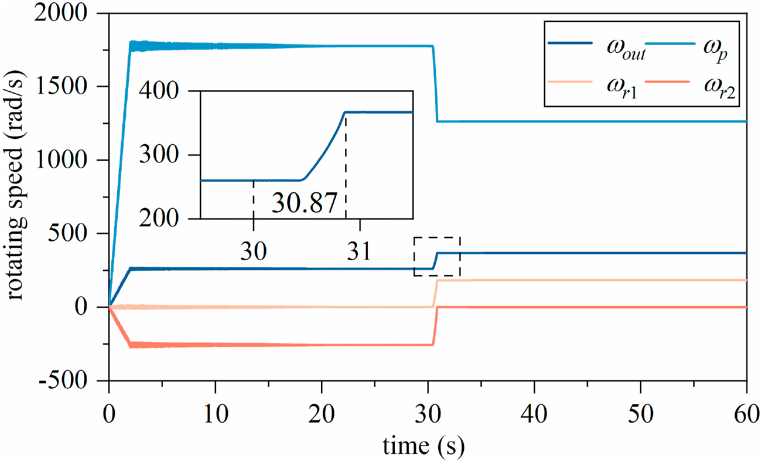
Time-domain curves of the whole shift process.

The simulation takes the XH-59A composite helicopter as a sample, referring to the performance parameters of the Pratt & Whitney PT6T-3 turboshaft engine with an output speed of 6000 rpm and a delivered power in the 1000 kW class. The compound dynamic model is solved by employing Runge–Kutta method, and the rotating speeds of the parts and the torque time-domain curve acting on the parts are obtained. Fig. 6 shows the rotating speeds of parts from start-up to upshift and to a stable state for a high gear. On commencing the upshift process after 30 s, after a brief delay, the upshift process is completed in approximately 30.87 s. Focusing on the phenomenon of rotating speeds delaying the response during upshifting.

**Fig. 7:**
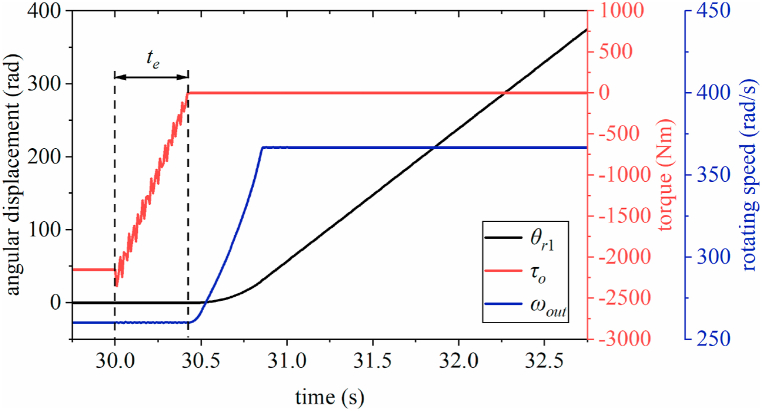
Time-domain curves at the beginning of upshifting.

Fig. 7 shows that at the beginning of the shifting process, the torque curve of the one-way clutch begins to oscillate, decreases to zero after *t*_*e*_. The angular displacement *θ*_r1_ of ring gear R1 does not change significantly during *t*_*e*_, and the output shaft speed *ω*_*out*_ remains stable at 260.2 rad/s. Subsequently, the one-way clutch is in the overrunning condition and no longer transmits the torque; *θ*_r1_ increases linearly, whereas *ω*_*out*_ gradually increases and then finally stabilizes at approximately 366.8 rad/s. The abovementioned results indicate that the torque imparted by the friction clutch to the compound planetary gear system does not lead to the one-way clutch full disengagement, at the beginning of the upshift. In other words, there is a “trade-off relationship” between the torque of two clutches.

#### 4.2.1 Effect of external incentives on shift dynamic

**Fig. 8:**
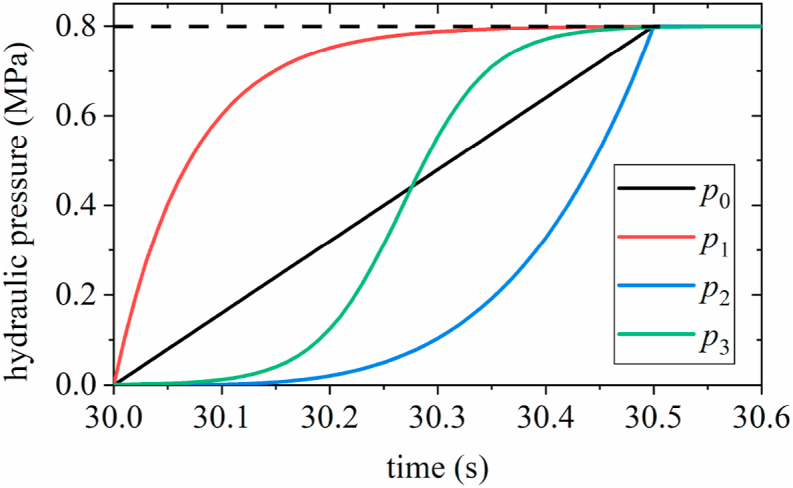
Curves of four hydraulic loading modes.

**Fig. 9:**
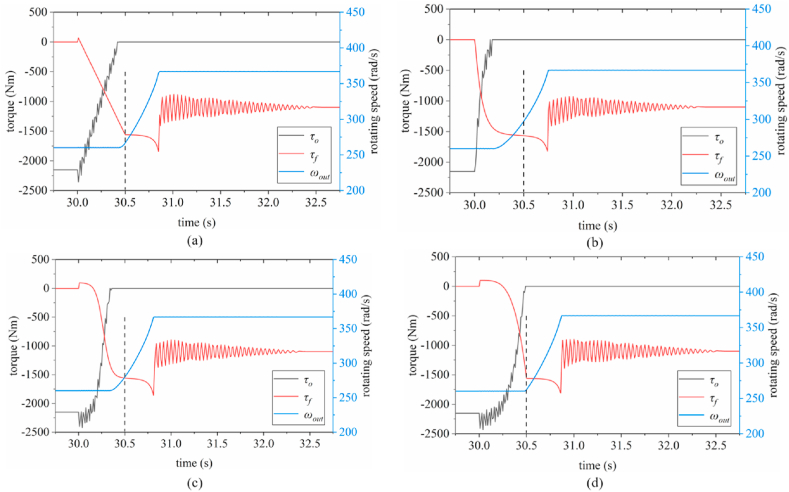
Time-domain curves of one-way clutch torque, friction clutch torque, and hydraulic pressure (a) linear function; (b) exponential function; (c) S-curve; (d) composite function.

The upshift characteristics of the TSDC transmission are impacted by the changing trend of engagement hydraulic pressure. In this study, in addition to the linear hydraulic loading mode *p*_0_, three additional hydraulic loading curves were designed based on the requirement of the loading hydraulic pressure reaching nominal hydraulic pressure in 0.5 s. The mathematical expressions for the four hydraulic loading curves are shown in Equations (8)–(11) [31,32].

(8) $$p_{0}=2P_{a}t$$

(9) $$p_{1}=P_{a}/\left(1-\exp\left(-14t\right)\right)$$

(10) $$p_{2}=P_{a}/\left(1+0.5\;\exp\left(6-25t\right)\right)$$

(11) $$p_{3}=16P_{a}t^{4}$$

where *t* is the hydraulic loading time. The pressure-characteristic curves are presented in Fig. 8. And the change curves of the hydraulic pressure, one-way clutch torque, and friction clutch torque during the shifting process were obtained, as shown in Fig. 9(a), (b), (c), and (d), respectively.

By comparing the time-domain curves in Fig. 9, it can be seen that except for Fig. 9(a), the torque of the other friction clutches fluctuates at the initial moment of the shift. and combined with the response of the one-way clutch, it can be found that the friction clutch torque jitter occurs simultaneously with the jitter of the one-way clutch torque. Observing the change trend of the four hydraulic loading curves, the exponential curve has a larger slope at the initial moment and the ramp-up process is more rapid compared to the other three curves. The difference of the above jitter can be considered as, the slow loading of hydraulic pressure at the initial moment of gear change causes the active end of the friction clutch to be dragged backward by the driven end, which indirectly leads to the torque jitter of the one-way and is transferred to the secondary inner gear ring through the wheel train, causing transient torque fluctuation of the friction clutch. In contrast, fast hydraulic loading can effectively avoid the active end being dragged and achieve a fast and stable engagement of the master and slave ends.

Fig. 9(a) shows that the torque of the friction clutch *τ*_*f*_, exhibits a small fluctuation, with a peak value of approximately 66.84 Nm. Subsequently, the *τ*_*f*_ increases, while the torque of the one-way clutch *τ*_*o*_, decreases to zero. At 30.5 s, the hydraulic pressure was loaded to the *P*_*a*_ and then kept constant; the *τ*_*f*_ changed nonlinearly, reached a peak value of 1841 Nm at 30.85 s and the speed of the output shaft *ω*_*out*_ reaches 366.8 rad/s at 30.86 s.

Fig. 9(b) indicates that the *τ*_*f*_ shows no fluctuations at the initial time, while the *τ*_*o*_ decreases rapidly to zero. At approximately 30.5 s, the *τ*_*f*_ and *ω*_*out*_ exhibit a steady state for a short period. The peak torque of the *τ*_*f*_ reaches 1819 Nm at 30.74 s. Moreover, the speed of the output shaft *ω*_*out*_ reaches 366.8 rad/s at 30.75 s.

As shown in Fig. 9(c), the *τ*_*f*_ exhibits a small fluctuation during the initial period, and the peak value is approximately 97.3 Nm. Here, the *τ*_*o*_ fluctuates and then decreases rapidly to zero. The *τ*_*f*_ reaches a peak value of 1857 Nm at 30.81 s, and the *ω*_*out*_ reaches 366.8 rad/s at 30.82 s.

Fig. 9(d) shows that the peak value of the *τ*_*f*_ is 99.67 Nm. A sudden change in the slope of *τ*_*f*_ was noted after 30.5 s. At 30.86 s, *τ*_*f*_ reaches a peak value of 1810 Nm. The *ω*_*out*_ reaches 366.8 rad/s at 30.87 s.

Table 3 presents a comparison of the change characteristics of two clutches torque curves under the four hydraulic loading modes during upshift.

**Table 3:** Torque variation of two clutches under four hydraulic loading modes.

Hydraulic loading modes	Law of torque curve change	
	Friction clutch	One-way clutch
*p*_0_	Fluctuations occur at the beginning of upshift, increasing linearly from 30 s to 30.5 s; the slope changes abruptly at 30.5 s. Peak value: 1841 Nm.	Approximately linear decrease, accompanied by vibration
*p*_1_	No fluctuations noted at the beginning of upshift, nonlinear increase, and no change in slope. Peak value: 1819 Nm.	Nonlinear rapid descent, with vibrations in the later stage of upshift
*p*_2_	Fluctuations noted at the beginning of upshift, nonlinear increase, and no change in slope. Peak value: 1857 Nm.	Nonlinear decrease, with clear vibrations during the early stage of upshift
*p*_3_	Fluctuations noted at the beginning of upshift, nonlinear increase, and slope changes abruptly at 30.5 s. Peak value: 1810 Nm.	Nonlinear decrease, with clear vibrations during the early stage of upshift

Based on the analysis results presented above, the *τ*_*f*_ under the *p*_1_ mode does not fluctuate during the initial stages of upshift, and the peak value of the *τ*_*f*_ is lower (*τ*_*f_max*_ = 1819 Nm), which is only 0.5 % higher than that in the *p*_3_ mode (*τ*_*f_max*_ = 1810 Nm). Moreover, the torque response of the one-way clutch under the *p*_1_ mode was faster, and the *ω*_*out*_ reached 366.8 rad/s within the shortest time (0.75 s); therefore, the *p*_1_ mode was deemed as the best among the four hydraulic loading modes considered.

#### 4.2.2 Gear shift duration

**Fig. 10:**
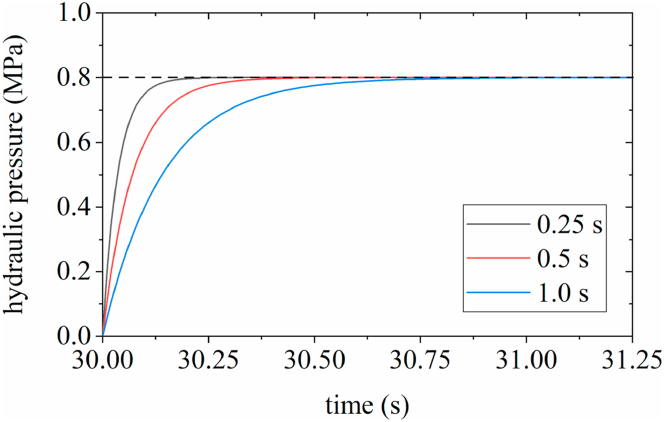
Three exponential hydraulic curves.

**Fig. 11:**
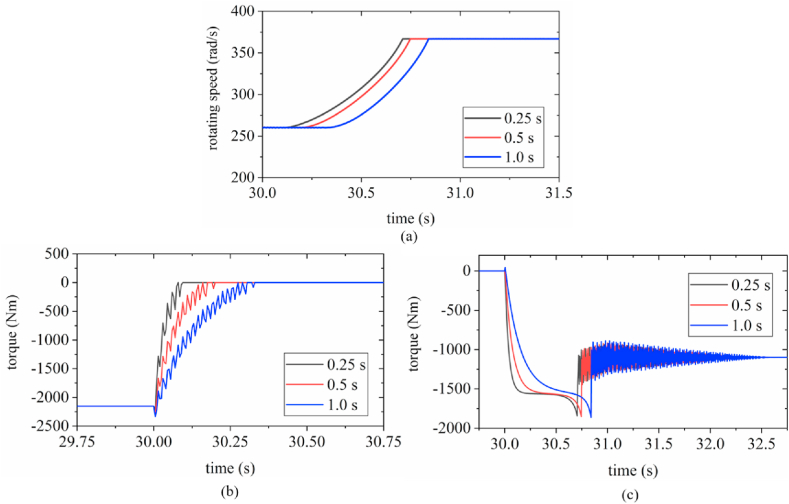
Time-domain curves (a) output shaft speed; (b) one-way clutch torque; (c) friction clutch torque.

The upshift duration impacts the output shaft speed, one-way clutch torque, and friction clutch torque. Assuming the other parameters remain constant, three types of exponential hydraulic characteristic curves, as shown in Fig. 10, are set. The hydraulic pressure reaches 0.8 MPa in 0.25, 0.5, and 1.0 s respectively. The simulation results are presented in Fig. 11.

As shown in Fig. 11(a), under the hydraulic loading modes of 0.25, 0.5, and 1.0 s, the output speeds reach stable values at a high gear at 30.72, 30.75, and 30.85 s, respectively. There is no significant difference among the trends of the output speeds under the three types of hydraulic loading; these trends indicate a nonlinear increase with no evident vibrations.

Fig. 11(b) shows that under the hydraulic loading modes of 0.25, 0.5, and 1.0 s, the one-way clutch is in a complete overrunning state at 30.10, 30.2, and 30.33 s. The one-way clutch torque *τ*_*o*_ under the three types of hydraulic loading exhibits a nonlinear decreasing trend, accompanied by clear fluctuations.

As shown in Fig. 11(c), the shorter the hydraulic loading process, the faster is the increase in the friction torque *τ*_*f*_ and the longer is the intermediate stable period in the shifting process. Under the hydraulic loading modes of 0.25, 0.5, and 1.0 s, the peak of the *τ*_*f*_ are 1842, 1852, and 1863 Nm at 30.705, 30.754, and 30.84 s, respectively. On extending the pressurization time, a corresponding delay is observed in the time of the peak torque. The *τ*_*f*_ under the three types of hydraulic loading reaches its peak value and then decreases rapidly.

## 5 Conclusion

A multi-component coupling model of the TSDC transmission system was constructed for the requirements of control design, including a composite planetary gear system bond graph model and two clutch torque models. Compared with the existing clutch-gear modeling method, the multi-system bond graph model features better flexibility and expansibility.

The simulation results indicate a delay in the speed response during the upshift process, which results from the trade-off relationship between the torques of two clutches.

Analyzed the influence of four types of hydraulic loading characteristics on the dynamic response during upshift, concluded that TSDC transmission can shift to a higher gear more smoothly and quickly under a load following an exponential function, as compared to that under the other types of loading.

It was also explained how the dynamics response was affected by the hydraulic loading speed. The faster the pressurization process, the faster the one-way clutch switches to a fully disengaged state, and the longer the friction clutch is in the middle state, and it does the time required to reach the peak torque. Future works will focus on the influences of load changes, gear backlash, and friction on the shift dynamics.

## Data availability

Data included in article/supp. material/referenced in article, data associated with this study has not been deposited into a publicly available repository.

## Funding statement

This work was supported by the 10.13039/501100001809National Natural Science Foundation of China (Grant No. 52075552).

## CRediT authorship contribution statement

**Jiangming Wu:** Methodology, Validation, Conceptualization. **Hongzhi Yan:** Formal analysis, Investigation. **Shuangqi Liu:** Formal analysis, Software. **De Ni:** Validation.

## Declaration of competing interest

The authors declare that they have no known competing financial interests or personal relationships that could have appeared to influence the work reported in this paper.
